# Magnetostatic Field System for Uniform Cell Cultures Exposure

**DOI:** 10.1371/journal.pone.0072341

**Published:** 2013-08-20

**Authors:** Cristian Vergallo, Claudia Piccoli, Alberto Romano, Elisa Panzarini, Antonio Serra, Daniela Manno, Luciana Dini

**Affiliations:** 1 Department of Biological and Environmental Science and Technology, University of the Salento, Lecce, Italy; 2 Department of Material Science, University of the Salento, Lecce, Italy; National Research Council, Italy

## Abstract

The aim of the present work has been the design and the realization of a Magnetostatic Field System for Exposure of Cell cultures (MaFiSEC) for the uniform and the reproducible exposure of cell cultures to static magnetic fields (SMFs) of moderate magnetic induction. Experimental and computer-simulated physical measurements show that MaFiSEC: i) generates a SMF with magnetic induction that can be chosen in the range of 3 to 20 mT; ii) allows the uniform SMF exposure of cells growing in adhesion and in suspension; iii) is cheap and easy to use. The efficacy and reproducibility of MaFiSEC has been tested by comparing the biological effects exerted on isolated human lymphocytes by 72 h of exposure to a magnet (i.e. Neodymium Magnetic Disk, NMD) placed under the culture Petri dish. Lymphocytes morphology, viability, cell death, oxidative stress and lysosomes activity were the parameters chosen to evaluate the SMF biological effects. The continuous exposure of cells to a uniform SMF, achieved with MaFiSEC, allows highly reproducible biochemical and morphological data.

## Introduction

The very fast production and diffusion of electric machineries, e.g., diagnostic and therapeutic medical apparatuses, mobile communications, domestic devices, etc., and their broad applications cause the continuous exposure of living organisms to electric and/or magnetic fields. The effective knowledge of the possible effects, negative or positive, on human health ignites the scientific community interest to deeply investigate the response to fields. In fact, a plethora of studies have been performed to understand not only the biological effects of electric and/or magnetic fields [1]–[3] but also the potential novel therapeutic use [4], [5].

However, literature data are contradictory [6], [7] and the interaction mechanisms between magnetic fields and cells, tissues and organisms are not fully elucidated and established [8]–[11]. Indeed, the experimental procedures, magnetic induction (ranging between 10^−7^ and 10 T), type of field (static or oscillating), type of biological samples used (i.e., molecules, cells and living organisms), etc. [12]–[14] are largely heterogeneous, thus they induce a negative influence on the reproducibility of the experimental results. Consequently, it is very important to made exposure systems able to minimize the variability of experimental set up.

Herein, we report data about a novel apparatus that could improve the spatial uniformity of Static Magnetic Field (SMF) applied to the cell cultures, ensuring the reproducibility of results. Our apparatus, named Magnetostatic Field System for Exposure of Cell cultures (MaFiSEC), is composed of two Neodymium (NdFeB) parallel rectangular magnets separated by air as dielectric medium. The reproducibility of data and the efficacy of this system were studied on isolated human lymphocytes, an experimental model already used in our previous studies about biological effects of SMF of moderate magnetic induction produced by a free magnet directly positioned under the culture dish (Neodymium Magnetic Disk, NMD) [12], [15]–[18]. Indeed, the optimal spatial uniformity of exposure that characterize MaFiSEC is an essential prerequisite of efficacy exposure reliability. MaFiSEC is an easy-to-use and efficacious tool for the study of the biological effects of SMF exposure.

## Materials and Methods

### MaFiSEC and NMD

**Figure 1:**
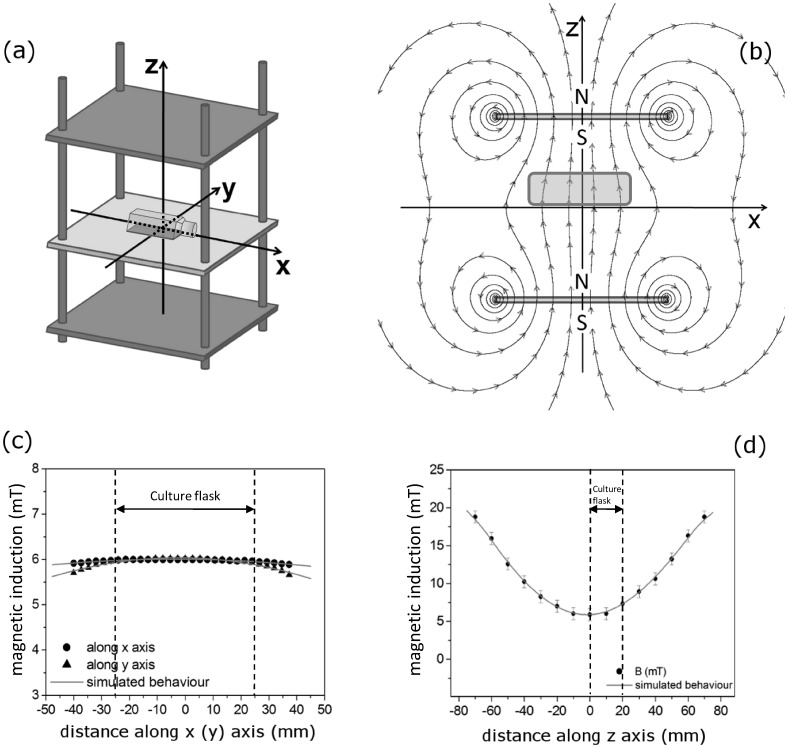
Exposure to SMF by using the Magnetostatic Field System for Exposure of Cell cultures (MaFiSEC). Schematic representation, not in scale, of (a) culture flask SMF-exposed with the MaFiSEC. The centre of the bottom of the culture flask has been designated as the origin ‘0’ of the reference system; the x and y axes were arbitrarily chosen, and the z-axis is perpendicular to flask and outgoing up from the same flask. The magnetic field is schematically represented by magnetic field lines, showing the direction of the field at different points (b). The filed lines crossing the sample are representative, schematic, i.e., they do not refer to measures of magnetic flux actually measured. (c, d): magnetic induction measured (spots) and simulated (continuous grey line) along (c) the flask and (d) in the perpendicular direction respectively. Error bar represents the SE of six independent experiments, each done in duplicate.

In MaFiSEC, SMF was produced by magnetic rectangular plates of NdFeB, sized 135×100×2 mm, coated with Ni, grade N35, *B_r_* 1170–1220 mT, magnetized through the thickness, supplied by China Rare Earth Magnets (Shenzhen, China). These magnets are mounted with opposite polarity in a structure of inert material to obtain a uniform SMF throughout the thickness of the cell suspension. The support structure was built using three synthetic polymers: i) plexiglass is the material used as support for the cell culture flask 2.5×10^3^ mm^2^ (central plane); ii) the lashings of the plans are made of nylon-66; iii) the housings of the magnets are made of PolyVinyl Chloride (PVC). These materials do not disturb the magnetic field configuration and are transparent to UltraViolet (UV) rays, that are used to sterilize MaFiSEC for cell culture. An additional iron plate, sized 150×110×1.5 mm, was inserted between the PVC and the magnets, with the functions to anchor the magnet to the PVC. The distance of the culture flask from magnets is not fixed, due to the presence of screws; thus the possibility to modify this distance allows to obtain magnetic fields of different inductions inside the cell cultures. The magnetic induction of SMF can be chosen in the range of 3–20 mT by changing the distance of magnets from the culture flask (Fig. 1a).

**Figure 2:**
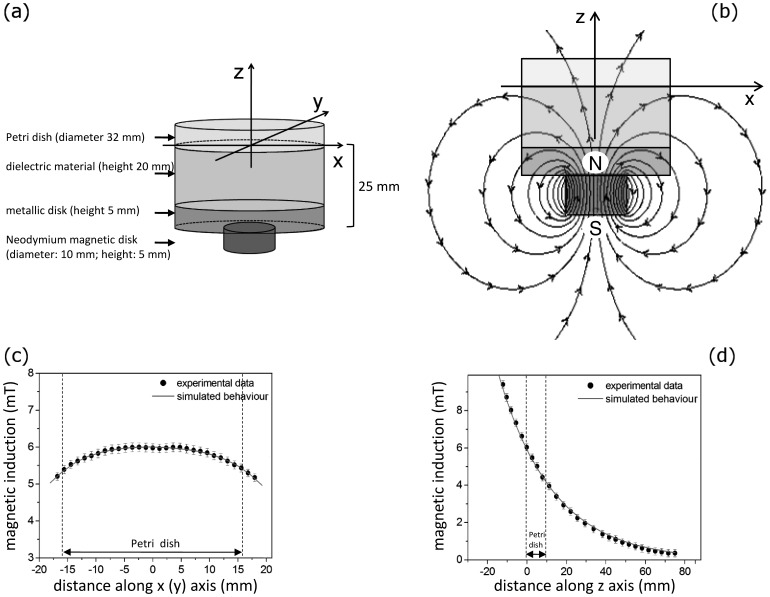
Exposure to SMF by using a Neodymium Magnetic Disk (NMD). Schematic representation, not in scale, of (a) Petri dish SMF-exposed with a NMD. The centre of the bottom of the Petri dish has been designated as the origin ‘0’ of the reference system; the x and y axes were arbitrarily chosen, and the z-axis is perpendicular to Petri dish and outgoing up from the same Petri dish. The magnetic field is schematically represented by magnetic field lines, showing the direction of the field at different points (b). The field lines crossing the sample are representative, schematic, i.e., they do not refer to measures of magnetic flux actually measured. (c, d): magnetic induction measured (spots) and simulated (continuous grey line) along (c) the Petri dish and (d) in the perpendicular direction respectively. Error bar represents the SE of six independent experiments, each done in duplicate.

In NMD, SMF was produced by a magnetic disk (diameter: 10 mm; height: 5 mm) of neodymium, grade N35, *B_r_* 1170–1220 mT, magnetized through the thickness, of known induction supplied by Calamit (Milan, Italy) placed under the culture Petri dish (diameter 32 mm) at a distance of 25 mm from the culture Petri dish (Fig. 2a).

### Measurements and Computer Simulations of Magnetic Induction

Analytic calculations has been performed in the approximation of uniform magnetization of the magnet properties by using the finite difference methods. In this way an ideal B-H characteristics applied to SMF and MaFiSEC exposure systems has been determined by Ansoft Maxwell 3D software (Ansoft, Pittsburgh, PA, USA). Our B-H curves confirm those of suppliers. Schematic, not in scale representation of the magnetic field lines, showing the direction of the field at different points of the culture flask inside MaFiSEC or of the Petri dish put above NMD, are shown in figure 1b and 2b respectively.

Magnetic field induction was measured using a digital gaussmeter (Model CI-6520A; PASCO Scientific, Roseville, CA, USA) with a sensitivity range of ±10 mT, 5 µT of resolution and 1 mT of accuracy, connected to a graphic interface (Science Workshop 750 interface; PASCO Scientific) (Fig. 1c–d and Fig. 2c–d, spots) and compared to the theoretical simulation. Indeed, by using the Ansoft Maxwell 3D software, we simulated also the magnetic induction along the width and the thickness of a culture flask or Petri dish exposed with MaFiSEC or NMD respectively (Figs. 1c–d and Figs. 2c–d, continuous line).

### Chemicals

All chemicals were of analytical grade and otherwise indicated were purchased from Sigma-Aldrich (Sigma, St. Luois, MO, USA).

### Ethics Statements

Human blood samples were obtained by buffy coats supplied by the Hospital S. Giuseppe da Copertino, Lecce, Italy. Donors were anonymous to us. The need of donor consent was waived by the Ethics Committee. The use of buffy coat was acknowledged by the Comitato Etico dell’ASL LE, Lecce, Italy (Ethics Committee of the Health Service of Lecce). This Ethics Committee is an independent organization that is working under the Declaration of Helsinki and following the rules of Good Clinical Practices according to international and national laws and to the guidelines of the Italian National Committee of Bioethics.

### Cells and SMF Exposure

Peripheral blood mononuclear cells were obtained after Ficoll gradient separation of buffy coats from blood donations of non-smoker healthy males, aged 25–45. Over 95% pure peripheral blood lymphocytes were separated from monocytes by double adherence to plastic. Lymphocytes were cultured in 25 cm^2^ flasks or 8 cm^2^ Petri dishes (Iwaki, Tokyo, Japan) at the same cell density of 10^6^ cells/ml in RPMI-1640 medium (Cambrex BioScience, Verviers, Belgium) supplemented with 10% (v/v) inactivated fetal calf serum (FCS) (Cambrex BioScience), 2 mM L-glutamine (Cambrex BioScience), 100 IU/ml penicillin and streptomycin in a humidified atmosphere of 5% CO_2_ at 37°C. Lymphocytes were used in all experiments 24 h after isolation, that was considered as time 0 (T0). Flasks containing 15×10^6^ lymphocytes and Petri dishes containing 5×10^5^ lymphocytes were exposed continuously for up to 72 h to SMF generated by MaFiSEC and NMD respectively. Control unexposed lymphocytes were cultured as above reported by using Petri dishes or flasks to exclude any influence of the different cell containers. Biochemical and morphological investigations were done at fixed interval times (24, 48 and 72 h) during continuous exposure to 6 mT SMF.

### Biochemical and Morphological Assays

Cell viability was evaluated by exclusion of vital dye Trypan Blue and by 3-(4,5-dimethylthiazol-2-yl)-2,5-diphenyltetrazolium bromide (MTT) assay. Generation of cytoplasmic ROS and lysosomal activity were evaluated by Nitro Blue Tetrazolium (NBT) and Neutral Red (NR) assays respectively.

#### MTT assay

The percentage of viable cells was indirectly determined by MTT dye reduction. Indeed, MTT is reduced by active mitochondria in living cells [19]. MTT assay was performed according to the modified method firstly described by Sladowski et al. in 1993 [20]. Briefly, 5×10^5^ lymphocytes were incubated with 1 mg/ml of MTT, prepared in supplemented RPMI-1640 culture medium, for 2 h at 37°C and 5% CO_2_; cells were then washed three times with phosphate buffer saline (PBS 0.2 M, pH 7.4) and the reduced MTT formazan crystals were solubilised with dimethyl sulfoxide (DMSO) (Carlo Erba, Milano, Italy). The optic density (OD) was read at the spectrophotometer (Ultrospec 4000 UV/Visible Spectrophotometer, Pharmacia Biotech, Stockholm, Sweden) at 570 nm.

#### NBT assay

The cytoplasmic nicotinamide adenine dinucleotide phosphate (NADPH), which is produced by oxidation of glucose through the hexose monophosphate shunt, serves as an electron donor [21]. The oxidase system available in the cytoplasm helps to transfer electrons from NADPH to NBT and reduces NBT into formazan [21]. Thus, the NBT reaction indirectly reflects the ROS-generating activity in the cytoplasm of cells. Briefly, 5×10^5^ lymphocytes were incubated with 335 µg/ml NBT, prepared in supplemented RPMI-1640 culture medium, for 2 h at 37°C and 5% CO_2_, then washed three times with absolute methanol (Carlo Erba). The amount of diformazan salts, dissolved with freshly prepared 2 M KOH (Azienda Chimica E Farmaceutica, Piacenza, Italy)/DMSO solution (460 µl KOH and 540 µl DMSO), was determined with a spectrophotometer at 630 nm.

#### NR assay

The NR uptake assay provides an estimation of the number of viable cells. It is based on the ability of viable cells to incorporate and bind the supravital dye NR into lysosomes [22]. Briefly, cells were incubated for 2 h at 37°C in a 5% CO_2_ moist atmosphere, with 0.01% (w/v, in culture medium) NR solution. After rinsing with PBS, the cells were added with the extraction solution (1% glacial acetic acid, 50% ethanol, 49% distilled water). The OD was read with the spectrophotometer at 540 nm.

#### Cell shape

Light microscopic (LM) analysis of lymphocytes, fixed with 4% formaldehyde (v/v, in PBS 0.2 M, pH 7.4) and haematoxylin-eosin (H-E) stained, allowed to score the morphological modifications induced by the SMF exposure. For each sample at least 500 cells in no less than 10 randomly selected fields were counted. Cells that showed an elongate shape as well as a ruffled surface or that showed morphological modifications of apoptosis (i.e., pyknotic nucleus, condensed chromatin, cell surface blebs) were on the whole classified as modified cells.

### Statistical Analysis

Three groups of data were compared, i.e. control *vs* NMD-exposed lymphocytes, control *vs* MaFiSEC-exposed lymphocytes and NMD- *vs* MaFiSEC-exposed lymphocytes by performing one-way ANalysis Of VAriance (ANOVA) at the 95% confidence level. A *post hoc* Bonferroni test was performed by keeping the experiment-wise error rate at 0.05 and by setting an adjustment factor of 3. Thus differences were considered to be significant at Bonferroni-adjusted critical p-value (indicated for simplicity with p) <0.0167 (0.05/3). The error bars represent the Standard Errors (SEs) of six independent experiments each done in duplicate. No significant differences were observed between control lymphocytes cultured by using either flasks or Petri dishes, thus the ‘control value’ corresponds to the average of values of unexposed cells irrespective of the culture container.

## Results

### Experimental and Computer-simulated Physical Measurements

With MaFiSEC, SMF was produced by magnetic rectangular plates of NdFeB organized as shown in figure 1a and reported in the Materials and Methods section. The induction of SMF can be chosen in the range of 3–20 mT. In the present work, the magnetic induction chosen for the experiments was 6 mT and was obtained at a 80 mm distance from the magnets. Figures 1c–d report the magnetic induction measured (spots) and simulated (continuous grey line) along the three directions of space, choosing as ‘0’ the center of the bottom of the flask (the x and y axes reside on the same plane, therefore, for convenience it has been reported only the behavior along the x axis). Figure 1b shows the magnetic flux pretty uniform. Indeed, relative to the central plane where the culture flask is located, a constant and uniform magnetostatic field with a magnetic induction of 6 mT is produced (Figs. 1c–d).

NMD was placed under the culture Petri dish at a distance of 25 mm in order to have 6 mT SMF right in the middle of the Petri dish. This 25 mm distance was obtained by interposing between the magnetic disk and the dish, a 5 mm metallic disk (to minimize the differences in the magnetic induction across the whole bottom of the dish) and a 20 mm thick inert disk (Fig. 2a). Magnetic field is represented by magnetic field lines and the arrows show the direction of the field (Fig. 2b). The field lines crossing the sample are representative, schematic, i.e., they do not refer to values of magnetic flux experimentally measured. A good correlation between the estimated and experimentally measured magnetic inductions was found. Magnetic induction measured (spots) and simulated (continuous grey line) along the bottom of the Petri dish (x and y axes) and along the z-axis are reported in figure 2c and d respectively.

In detail, NMD allows to achieve a uniform 6 mT SMF within the range of –10≤0≤ +10 mm along the axes x, y and of –1≤0≤ +1 mm along the z-axis (Figs. 2c–d), corresponding to a total volume of 4×10^2^ mm^3^. Conversely, MaFiSEC allows to obtain a uniform 6 mT SMF within the range of –35≤0≤ +35 mm along the axes x, y and of –10≤0≤ +10 mm along the z-axis (Figs. 1c–d), corresponding to a total volume of 4.9×10^4^ mm^3^, which is more than 100 times greater than the volume exposed with NMD. In addition, due to the overlap of the two individual magnetic fields generated by the two magnets, MaFiSEC produces a magnetic flux denser than the one generated by the NMD, exactly where is the culture flask (please, compare Fig. 1b with Fig. 2b).

Lymphocyte cultures were always placed on the same two shelves in a cell culture incubator where the ambient 50 Hz magnetic field was 0.95/0.62 µT (heater on/off). In the experimental room where the physical measures were done, the background magnetic induction was 10 µT (static), while in the laboratory area where the cells were processed (between incubators, worktops and cell culture hood) the magnetic field measured ranged between 0.08 and 0.14 µT (50 Hz). The local geomagnetic field was approximately 43 µT; no other significant effect, including any temperature rise, was detected throughout 72 h. The errors for all measures never exceeded 2%.

### Biological Effects of NMD and MaFiSEC Exposure

**Figure 3:**
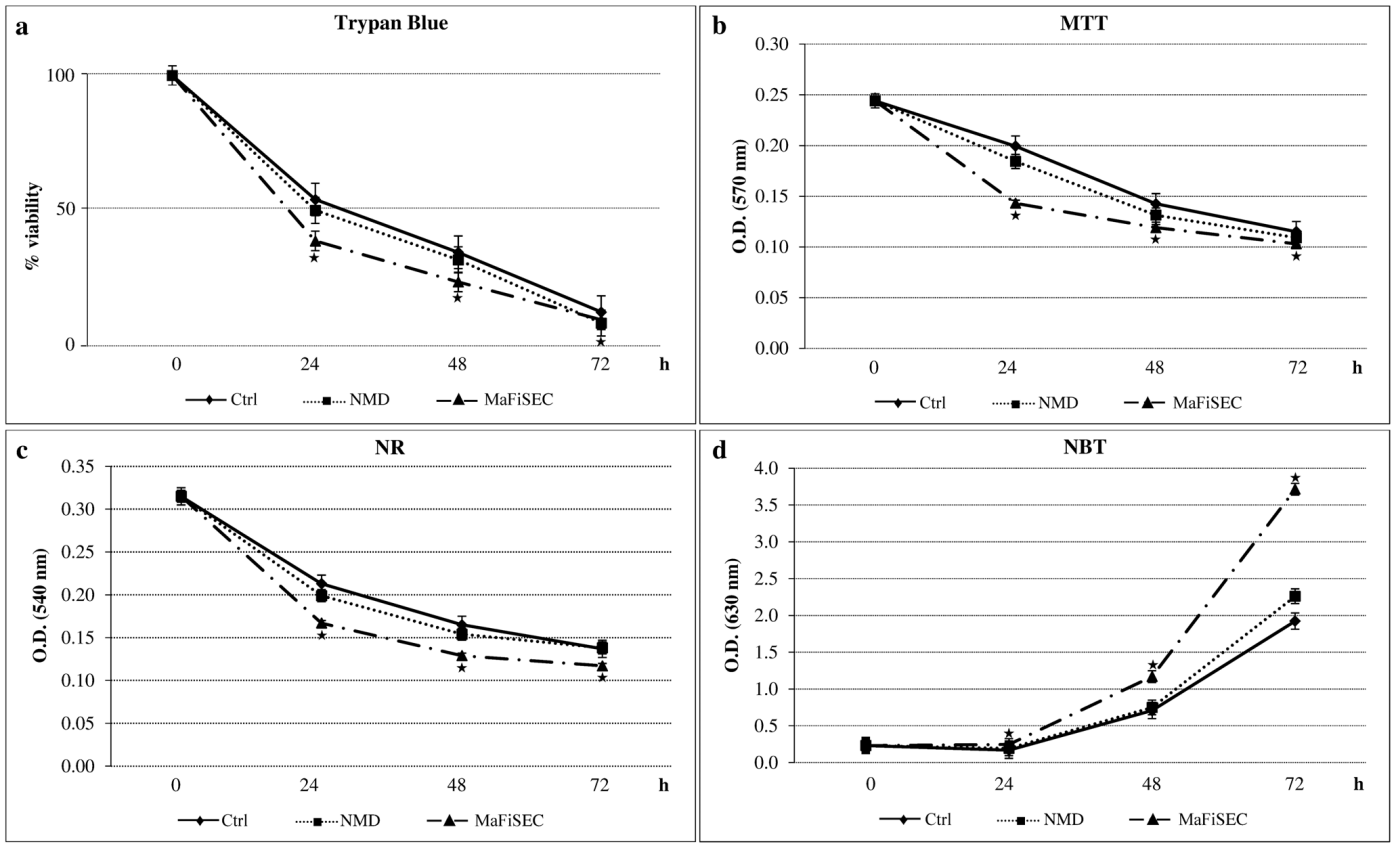
Time-course of SMF cytotoxicity. Viability of SMF-exposed lymphocytes: (a) Trypan Blue dye exclusion assay; (b) MTT assay; data are reported as percentage of viability of unexposed lymphocytes at T0, taken as 100%. (c) Lysosomal activity of SMF-exposed lymphocytes, NR assay. (d) ROS production by SMF-exposed lymphocytes, NBT assay. Error bar represents the SE of six independent experiments, each done in duplicate. Star indicates significant value with respect to unexposed and NMD-exposed cells (p<0.0167). Control value is the average of the values of unexposed cells cultured by using either flasks or Petri dishes. Ctrl = unexposed lymphocytes; O.D. = optical density; h = hours.

The effects of SMF exposure on viability (Trypan blue exclusion dye, mitochondrial and lysosomal activities), ROS production and cell shape of isolated human lymphocytes were analysed at 24, 48 and 72 h of continuous SMF exposure with NMD or MaFiSEC (Figs. 3a–d).

Viability of lymphocytes was largely affected by SMF exposure. Indeed, irrespective of the method of study (i.e., Trypan Blue, MTT or NR tests), MaFiSEC-exposed cells showed at all times of investigation a significant lower viability than unexposed control lymphocytes (p<0.0167, Figs. 3a–c). Conversely, the observed reduction of viable cells in the lymphocytes population exposed with NMD was moderate (Figs. 3a–c). Interestingly, the values between NMD- and MaFiSEC-exposed cells were always significant (p<0.0167). Thus, the two exposure systems induced both a decrease of cell viability but at a different extent. The knowledge that isolated human lymphocytes, in absence of mitogens, underwent to a progressive physiological cell death (i.e., spontaneous apoptosis) explains why the highest differences between control and SMF-exposed lymphocytes are found within the first 24 h of observations (Figs. 3a–b). In particular, viability of MaFiSEC-exposed lymphocytes dramatically decreased at 24 h of exposure (Figs. 3a–b).

Reduced cell viability is accompanied with increased ROS generation (about twice above control unexposed lymphocytes at 72 h) and lysosomes activities in MaFiSEC-exposed lymphocytes (Figs. 3c–d), thus suggesting a high toxicity. Conversely, levels of ROS and lysosomes activities during NMD exposure were never significant different from unexposed cells (Figs. 3c–d).

**Figure 4:**
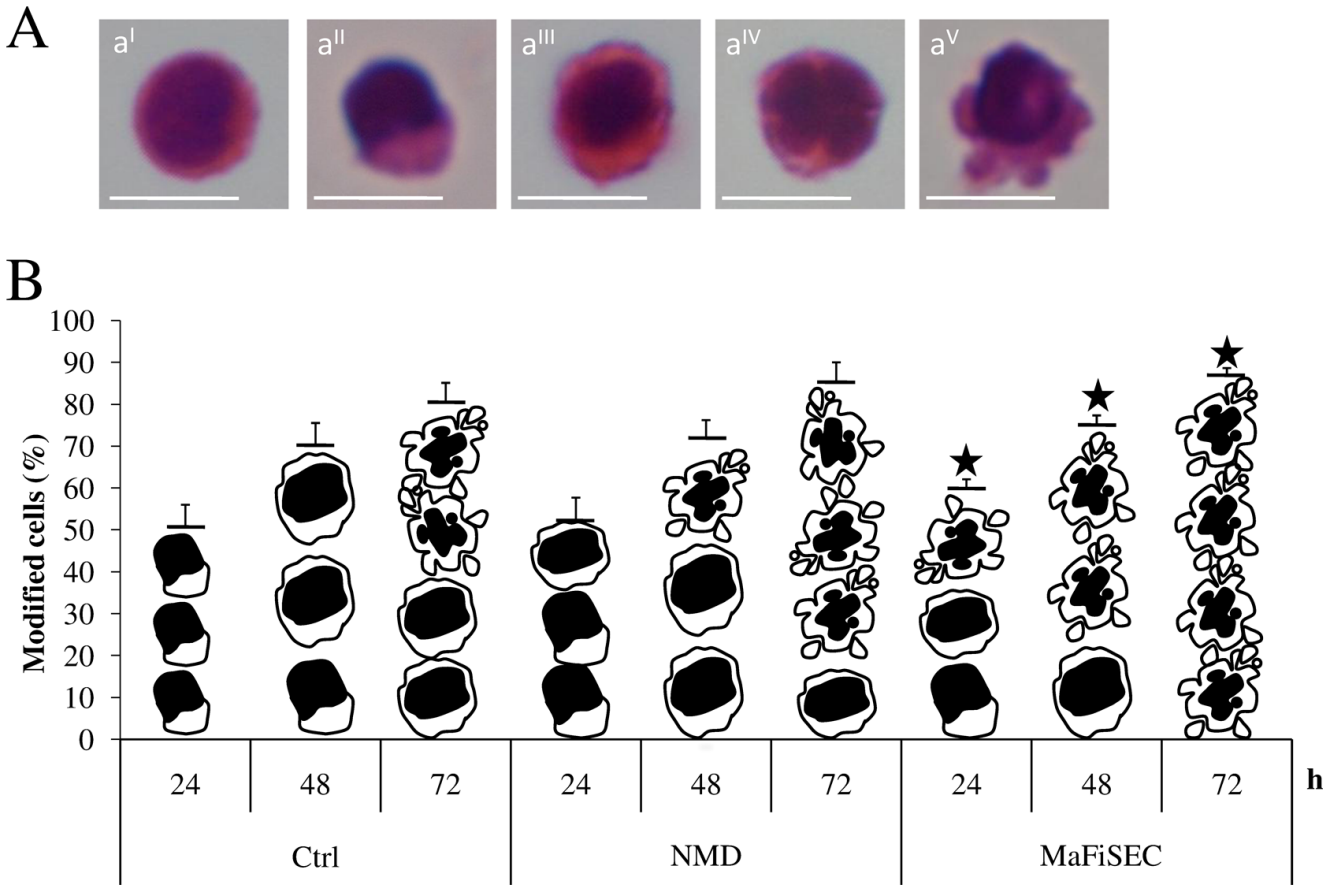
Morphologies of unexposed and SMF-exposed lymphocytes. (A): LM micrographs of H-E stained lymphocytes: typical normal (a^I^) and altered (a^II–V^) morphologies of unexposed and SMF-exposed lymphocyte respectively; (a^II^) an example of a lymphocyte with elongate shape; (a^III–V^) typical features of apoptosis: a lymphocyte with a pyknotic nucleus and rough surface (a^III^), condensed chromatin and fragmented nucleus (a^IV^) and extensive cell surface blebbling (a^V^). Bars = 10 µm. (B): schematic drawings of the different morphologies of lymphocytes, representing the pictures in A, show the percentage of incidences of the different modified morphologies during 72 h of SMF exposure to NMD and MaFiSEC. Each height of the histogram is the sum of the percentages of modified cells indicate at the lines 2–4 of the table 1. Control value is the average of the values of unexposed cells cultured by using either flasks or Petri dishes. Ctrl = unexposed lymphocytes; h = hours. Star indicates significant value with respect to unexposed and NMD-exposed cells (p<0.0167).

Morphological modifications of lymphocytes in culture are quite a common event, even in unexposed cells, partially as a result of the isolation mechanical stress and, largely, of the spontaneous apoptosis onset [12], [15]. In fact, about 50% of the unexposed lymphocytes shows a modified cellular shape (elongation, nucleus and cytoplasm blebbing, ruffled surface, etc.) already at T0 (Fig. 4a ^II^–b). From 24 to 72 h of culture, the morphological modifications are mainly related to apoptosis, i.e., pyknotic nucleus, condensed chromatin and cell surface blebs (Fig. 4a ^III–V^–b). The percentage of lymphocytes bearing apoptotic modifications increased with time of SMF exposure (Fig. 4b). However, MaFiSEC-exposed lymphocytes showed already at 24 h extensive nuclear and cytoplasmic blebbing with respect to NMD-exposed cells (Fig. 4b). Table 1 shows the percentages with the relative SEs of the different morphologies of lymphocytes during 72 h of exposure by using MaFiSEC or NMD.

**Table 1:** Percentages of normal or modified morphologies of lymphocytes during 72 h of culture in absence or presence of 6 mT SMF.

Shape	Ctrl				NMD			MaFiSEC			
	24	48	72 h		24	48	72 h	24	48		72 h
Normal (a^I^)	49,21±3,71	29,48±3,33		16,14±4,73	47,47±3,66	27,94±2,84	14,83±1,95	39,88±3,01		24,83±1,87	12,89±1,33
Elongated (a^II^)	50,79±5,24	23,51±1,67		–	35,02±3,45	–	–	22,04±0,67		–	–
Pyknotic nucleusand rough surface(a^III^–a^IV^)	–	47,01±3,34		39,42±1,18	17,51±1,72	48,04±2,79	21,29±1,21	18,03±0,66		25,06±0,82	–
Extensive cellsurface blebbling(a^V^)	–	–		44,44±3,09	–	24,02±1,39	63,88±3,62	20,05±0,65		50,11±1,35	87,11±2,55

a^I–V^ = cell morphologies as shown in Fig. 4A; Ctrl = unexposed lymphocytes; h = hours; NMD = Neodymium Magnetic Disk; MaFiSEC = Magnetostatic Field System for Exposure of Cell cultures; – = shape not detectable.

## Discussion

Cell cultures are often used as experimental model for the understanding of the biological effects of SMFs that might happen at organisms level including humans. It is, thus, strictly important to use a reliable cell culture exposure system. To this purpose we designed and built a novel system, i.e., MaFiSEC, for cell cultures SMF exposure to overcame the difficulties in the reproducibility of experimental data. Reliability of MaFiSEC was proved by theoretical and experimental physic measures and by biochemical and morphological analysis of lymphocytes. Biological effects were compared with those obtained by simply using a magnet under the culture Petri dish.

The efficacy of exposure to induce biological response was tested by choosing the known biological and biochemical activities modified by a SMF of moderate induction, as dictated by our and by others authors experience [18], [23]–[27].

MaFiSEC allows the high reproducibility of data, as suggested by low SEs, that are, in addition, smaller than SEs calculated with NMD. The MaFiSEC high reproducible data is very important, especially in those cases in which it is difficult to have many experimental replicates due to the biological sample shortage.

It is known that exposure to SMF interferes with cell viability and/or cell death and with the roundish shape of in suspension growing cells that are modified to an elongate or irregular cellular shape with increasing presence of microvilli [15]. Indeed, lymphocytes viability decreased while the number of lymphocytes bearing morphological alteration and the number of cell deaths increased. All these data suggests that the reduction and/or elimination of even small percentage of un-homogeneity of exposure, produces big cell responses differences. Consequently MaFiSEC allows to better verify the dose and the exposure time dependency. Most likely toxicity of MaFiSEC is driven by the increased production of ROS, that in turn, trigger apoptosis [28]–[30]. Indeed, MaFiSEC promotes a fast onset of apoptotic cells (see Fig. 4). In fact, the most frequent morphological modifications of lymphocytes after exposure to MaFiSEC were those typical of late stages of apoptosis, in particular, pyknotic nucleus, condensed chromatin and nuclear and cytoplasmatic blebs (Fig. 4a ^III–V^–b). MaFiSEC is, thus, suitable for the study of the apoptotic process induced by physical factors in lymphocytes.

By studying the biological effects exerted by exposure to SMF on different cell lines [12], [15], [17], [18], [31], we already shown that, beside the cell type, duration and induction of the exposure, were largely influenced by the continuity and uniformity of exposure. Even if NMD and MaFiSEC are based on magnets, these are different for size and shape. NMD magnet is a disk, smaller than the magnet of MaFiSEC that is a plate. However, the most relevant difference relays on the modality of the respective allocation of magnet and cells. In MaFiSEC, the magnets are fixed in a determined part of the structure, inert to SMF, while culture flask has a special allocation. The fact that the culture flasks are always placed in the same position allows the cells to be exposed always in the same manner, i.e. with respect to the uniform SMF produced by the central part of the magnet. This is undoubtedly an advantage. Conversely, since NMD is not inserted in a structure but it is a free magnetic disk, the reproduction of the exact position with respect to the cell culture dish is difficult.

A constrained system for the exposure of cell cultures was already proposed by other groups [32]–[34]. The advantage of our exposure system consists in the possibility to set the magnetic induction within a defined interval, characteristic not provided by the other systems. Indeed, MaFiSEC takes advantage of the adjustments that can be done within the limits of the system itself and the strength of the magnets. Thus, the range of variability of the magnetic induction is between 3 and 20 mT. In this range, irrespective of the chosen magnetic induction, the SMF is uniform.

We already experienced that a uniform exposure of cells growing in suspension, due to their continuous movement inside the culture medium, is more difficult to be obtained than exposure of in adhesion growing cells. Indeed, this is the reason why reliability of MaFiSEC was verified using isolated human lymphocytes. It turns out that, MaFiSEC offers a uniform field to the whole medium containing the cells. In fact, in our experiments 1.5×10^7^ lymphocytes, at a concentration of 10^6^/ml, occupied a total volume of 15 ml, that corresponds to 1.5×10^4^ mm^3^, that is 3.3 fold less than the total volume uniformly exposed by MaFiSEC, i.e., 4.9×10^4^ mm^3^. It is important to note that in MaFiSEC the SMF is uniform at the bottom of the flask and in the whole medium occupied by the floating lymphocytes suspension. Thus, MaFiSEC- but not NMD-exposed lymphocytes, are uniformly and continuously exposed, since they are never outside the SMF or in un-uniform SMF. This large volume control of SMF induction is, of course, suitable also for the exposure of adherent cells, in which vertical differences are absent.

The higher effects observed in lymphocytes exposed to MaFiSEC with respect to cell viability, ROS production and apoptosis was the result of the uniform and continuous exposure conditions. This, in turn, induced a faster and higher number of lymphocytes to enter the apoptotic program. In fact, a^III^ and a^IV^ categories were not observed in the lymphocytes population after 72 h of MaFiSEC exposure (second and third line of the ninth column in table 1).

In conclusion MaFiSEC is a very useful tool for the study of the biological effects of SMFs on different cell lines, for the uniform magnetic induction exposure, reproducibility of exposure (i.e., for the precise and fixed place of cell culture flasks) and, finally, for reliability due to the above characteristics, the data obtained are trustworthy. In addition MaFiSEC, due to its specific characteristics, is also cheap to build and easy to use in the range of 3 to 20 mT SMF induction. This will allow to get a better knowledge of the biological effects of SMF, no matter if positive or negative, that, in turn, will help to discriminate among possible adverse or therapeutic outcomes for human health.
